# Prevalence, risk factors, treatment uptake and treatment outcome of hepatitis C virus in people who inject drugs at the needle and syringe program in Uppsala, Sweden

**DOI:** 10.1186/s12954-023-00806-w

**Published:** 2023-06-16

**Authors:** E. Kågström, A. Lannergård, J. El Khosht, P. Lörelius, J. Månflod, S. Strömdahl

**Affiliations:** 1 Department of Medical Sciences, Infectious Diseases, Uppsala University, Uppsala, Sweden; 2 Needle and Syringe Program Uppsala, Nära Vård och Hälsa, Region Uppsala Uppsala, Sweden

**Keywords:** Hepatitis C, People who inject drugs, Needle exchange program, Harm reduction, Injection drug use, Injection risk behavior, Reinfection, Needle syringe program

## Abstract

**Background:**

The World Health Organization has set a goal to reach world elimination of hepatitis C virus (HCV) by 2030. Needle and syringe programs (NSP) for people who inject drugs (PWID) are crucial to achieve this goal. The NSP in Uppsala, Sweden, was opened in 2016 and has since 2018 provided HCV treatment for PWID. The aim of this study was to investigate HCV prevalence, risk factors and treatment uptake and outcome in NSP participants.

**Methods:**

Data from 450 PWID registered at the Uppsala NSP between 2016-11-01 and 2021-12-31 were collected from the national quality registry InfCare NSP. Data from the 101 PWID treated for HCV at the Uppsala NSP were collected through patient journal review. Descriptive and inferential analysis was performed. Ethical approval was obtained from the Ethical Review Board in Uppsala (dnr 2019/00215).

**Results:**

The mean age was 35 years. 75% were males (336/450), and 25% were females (114/450). The overall HCV prevalence was 48% (215/450) with a declining trend over time. Factors associated with a higher risk of HCV were older age at registration (OR 1.025, 95% CI 1.004–1.046), lower age at injection drug debut (OR 0.963, 95% CI 0.932–0.996), lower education level (OR 1.829, 95% CI 1.185–2.821) and higher number of total visits at the NSP (OR 1.005, 95% CI 1.001–1.009). The overall HCV treatment uptake was 47% (101/215), of which 77% (78/101) completed HCV treatment. The HCV treatment compliance was 88% (78/89). 99% (77/78) were cured with a sustained virologic response 12 weeks after completed treatment. The reinfection rate over the study period was 9/77 (11.7%); all were male with mean age of 36.

**Conclusions:**

HCV prevalence, treatment uptake and treatment outcome have improved since the opening of the Uppsala NSP. However, further measures are needed to reach the HCV elimination goal. Outreach HCV treatment programs for PWID should be explored and evaluated in combination with further implementation of low-threshold programs.

## Background

Hepatitis C virus (HCV) is a blood-borne virus that causes chronic liver inflammation, which over time leads to liver cirrhosis, liver cancer and liver-related death [1, 2]. The prevalence is estimated at 58–80 million people globally and the mortality at approximately 290,000 deaths in 2019 [3–5]. The most common transmission route for HCV in high-income countries is sharing of unsterile injection equipment (needle/syringes and/or paraphernalia) among people who inject drugs (PWID), making this subgroup subject for special attention [6, 7]. In Sweden, an estimated 80% of new HCV infections are caused by sharing of unsterile injection equipment among PWID [8].

The World Health Organization (WHO) has set a global goal to reach world elimination of HCV and hepatitis B virus (HBV) by 2030, which includes a 90% reduction in chronic infection and a 65% reduction in HCV-related deaths [9]. Available data show that although major progress has been made we are far from the 2030 goal with most countries not on track to reach the targets [5, 10]. An important service target to reach this goal is distribution of at least 300 sterile needles and syringes/PWID/year [9]. Needle and syringe programs (NSP) are harm reduction services that provide clean injection equipment and other health services to PWID. NSPs are a cornerstone to achieve the HCV elimination goal, as they contribute to the reduction of HCV transmission and injection risk behavior among PWID [11–13]. The number of NSPs in Sweden has increased dramatically in the last 10 years after a long period of political hesitancy. Following a change in the NSP law in 2017, a scale-up of NSPs in Sweden started [14]. In the end of 2020, 17 out of 21 regions had launched NSP [15]. The NSP in Uppsala opened in 2016 has since 2018 offered free HCV treatment to all patients.

Data regarding the size of the PWID population in Sweden have been estimated at between 8000 and 26,000; however, the data are outdated and inconclusive and estimation is hard due to methodological difficulties [12, 16–18]. Previous estimates have predicted the HCV prevalence in the PWID population in Sweden in 2016 at 60% and the treatment rate of HCV positive PWID in 2015/2016 to 0.5–2% [19]. A 2017 study from the NSP in Stockholm showed a HCV prevalence of 62.1%, but no such analysis has been performed on the PWID population in Uppsala [20]. HCV treatment is free in Sweden and consists of direct acting antivirals (DAA) [21, 22]. DAAs are an effective, well-tolerated and safe treatment with cure rates of > 95%; however, more data are needed on compliance and treatment results in the PWID population [23, 24]. An analysis from 2021 showed that achieving the WHO elimination goal in Sweden will require an expansion of harm reduction programs to reach 90% of the PWID population and treating 90% of HCV-positive NSP participants as well as > 7% of HCV-positive PWID not attending harm reduction programs [25]. To achieve this goal, an evaluation of the NSPs and its’ effects on HCV in the PWID population is needed.

The aim of this study is to examine HCV prevalence, risk factors, treatment uptake and treatment outcome among participants at the Uppsala NSP. These data are needed to estimate the effect of the NSP as well as identify areas in need of improvement.

## Methods

### The NSP in Uppsala

The NSP in Uppsala opened in 1 November 2016 is located in a primary care facility. In addition to offering exchange of needles, syringes and paraphernalia, it also provides vaccine for hepatitis A (HAV) and HBV, testing for HAV, HBV, HCV, human immunodeficiency virus (HIV) and other sexually transmitted diseases (chlamydia, gonorrhea, syphilis), treatment for HCV (initiated in 2018), treatment for injection-related infections and primary care. The patients are offered nasal naloxone along with a brief education in cardiopulmonary resuscitation. At the NSP there is a broad competence consisting of two clinical nurse specialists and a counselor working full-time as well as a general practitioner, a psychiatrist, a midwife and a substance abuse counselor working part-time. There is also an infectious disease consultant available.

### Study population and data

The study consists of two parts. First, an extraction of anonymous data from the national quality registry InfCare NSP of NSP participants in Uppsala during the study period was performed [26, 27]. Secondly, a patient journal review study was performed on longitudinal data from those treated for HCV at the Uppsala NSP who had provided an informed written consent.

### The NSP national quality registry

The NSPs in Sweden collaborate on the national quality registry InfCare NSP where sociodemographic data and HCV test result data are entered at enrollment and follow-up visits [26, 27]. Anonymous data were extracted from the NSP quality registry for all individuals attending the Uppsala NSP between 1 November 2016 and 31 December 2021 that had registered in the InfCare NSP. NSP participants are at the first visit informed of the national quality registry by trained health staff and provide an oral informed consent according to the standards set for the national quality registry [26, 27]. Participants are also informed that they can opt out at any time, and written information of this is available in the NSP waiting area.

The requirements to register at an NSP in Sweden are served as inclusion criteria for the national quality registry and this study; these are: age 18 years or above, documented injection drug use, valid identification and no current admission to a hospital or substance abuse treatment center. Participants registered at other NSP and then transferring to Uppsala NSP were included, making possible date of first registration as early as 2013. Patients with missing registration data and/or missing HCV test were excluded as well as patients also registered at other NSP in Sweden who were registered in Uppsala less than 1 month in total during the study period. At the NSP registration visit participants are tested for HBV, HCV and HIV and they also engage in a face-to-face interview with NSP staff and fill out a standardized questionnaire regarding birth date, home municipality, residence municipality, education level, marital status, living situation, employment, if they have children, status and previous testing for HIV, HBV and HCV, vaccine status, age of drug debut, first drug used, age of injection drug debut, first injected drug, main drug used currently, injection use past month, past injected drug, injection risk behavior, custody/prison time past year, being in opioid substitution treatment, being in substance use treatment center past year, being admitted to a hospital past year, condom use during sex past month and selling or buying sex. After this, follow-up interviews and HCV testing are performed every 6 months, if possible. Our analysis used the HCV data from registration and follow-up testing from the national quality registry. For participants with negative HCV RNA test at registration, either negative HCV RNA or negative anti-HCV test was considered negative HCV status at follow-up testing. In the dataset from the national quality registry, we only had information on the seroprevalence of anti-HCV on some participants; therefore, no analysis on anti-HCV for the study population is presented in our results.

### Patient journal review of NSP participants treated for HCV

The second part of the study consists of a patient journal review of the 101 NSP participants who have completed or started HCV treatment at the Uppsala NSP between 1 November 2016 and 31 December 2021.

For patients with positive HCV RNA, genotype analysis and risk of liver disease are determined. Patients at risk are examined with FibroScan. Patients with positive HCV status are then offered HCV treatment at the NSP. At the initial treatment visit at the NSP, the participants are given study information both orally and in writing by trained health staff. They then provide an informed written consent. Participants are also informed that they can withdraw from the study at any time.

Patient journal review was performed for the variables gender, age, liver status, virus type, virus level, way of transmission, date of transmission (when known), date of diagnosis, treatment received, complications from treatment, discontinued treatment, if SVR had occurred, if reinfection has occurred and mortality. An undetectable HCV RNA 12 weeks after therapy completion was considered a sustained virologic response (SVR).

### Immunological and virological methods

All virological tests for HCV RNA were performed using the Abbott Alinity m system, with a limit of detection of 12 IU/mL. All serological tests for HCV were performed using the Abbott Alinity i system, with a sensitivity of 100% (95% confidence interval of 99.2–100%).

### Statistical methods

The statistical program IBM SPSS 28.0.0.1 was used for all analyses. Descriptive analysis was performed for demographic variables and is presented as proportions, mean or median levels and range. The Chi-square test or Fisher exact test was used to test categorical variables.

Simple and multiple univariate logistic regression was used to study the associations with the outcome variable, positive HCV RNA status. Eleven possible determinants were tested against the outcome selected based on HCV risk factors reported in previous research; gender, age at registration, age of drug use debut, age of injection drug use debut, number of visits to the NSP during the study period, level of education, living situation the past 3 months, employment in the past 3 months, primary drug used the past 12 months, shared needle/syringe and/or paraphernalia in the past month and HBV status at registration [12, 28]. The results are reported as odds ratios (OR) with confidence intervals (CI) set at 95%. A *p* value < 0.05 was considered statistically significant.

### Ethics

Ethical approval for the study as described above was obtained from the Regional Ethical Review Board in Uppsala, Sweden, with dnr number: 2019/00215.

## Results

Of the 519 participants identified in the NSP national quality registry according to the inclusion criteria, 17 were excluded due to missing registration data, 15 due to missing HCV test and 37 due to less registration time than one month at Uppsala NSP resulting in 450 participants included in the analysis.

### Sociodemographic data

In Table 1 demographic data are presented in total and per HCV serostatus. Of the 450 included participants, 336 (74.7%) were male and 114 (25.3%) were female. The mean age at registration was 34.6. A majority was born in Sweden and had Uppsala as their home region, representing 310 (88.1%) and 283 (65.8%) participants, respectively. 42 patients were born outside of Sweden; 16 in Europe, 24 in Asia, 1 in Africa and 1 in South America. Of the 147 participants with other home region than Uppsala, the most common regions were the adjacent regions Gävleborg (42/147), Stockholm (32/147) and Västmanland (31/147).

**Table 1:** Baseline demographic data in total and related to HCV status

	All *n* (%)	HCV positive *n* (%)	HCV negative *n* (%)	*p* value
Total	450 (100)^a^	215 (100)	235 (100)	
Gender				0.750
Male	336 (74.7)	162 (75.3)	174 (74)	
Female	114 (25.3)	53 (24.7)	61 (26)	
Age at registration				N/A^b^
Mean age (years)	34.60	35.30	33.95	
Median age (years)	31	32	31	
Total number of NSP visits				< 0.001
1	41 (9.1)	17 (7.9)	24 (10.2)	
2–10	161 (35.8)	63 (29.3)	98 (41.7)	
11–100	207 (46)	105 (48.8)	102 (43.4)	
> 100	41 (9.1)	30 (14)	11 (4.7)	
Country of birth				0.486
Sweden	310 (88.1)	152 (86.9)	158 (89.3)	
Other	42 (11.9)	23 (13.1)	19 (10.7)	
Home region				0.01
Uppsala	283 (65.8)	121 (59.6)	162 (71.4)	
Other	147 (34.2)	82 (40.4)	65 (28.6)	
Age of debut of drug use				N/A
Mean age (years)	14.81	14.20	15.37	
Median age (years)	14	14	15	
Age of debut of injection drug use				N/A
Mean age (years)	22.45	21.10	23.70	
Median age (years)	20	19	21	
Level of education				< 0.001
Partial elementary	49 (11.2)	28 (13.5)	21 (9.1)	
Elementary	174 (39.8)	99 (47.8)	75 (32.6)	
Upper secondary	153 (35)	59 (28.5)	94 (40.9)	
Vocational	33 (7.6)	15 (7.2)	18 (7.8)	
University	28 (6.4)	6 (2.9)	22 (9.6)	
Living situation the past 3 months				0.009
Own housing	172 (38.5)	65 (30.5)	107 (45.7)	
Shared housing	116 (26)	59 (27.7)	57 (24.4)	
Homeless/Shelter	98 (21.9)	51 (23.9)	47 (20.1)	
Assisted living	20 (4.5)	11 (5.2)	9 (3.8)	
Treatment home	13 (2.9)	9 (4.2)	4 (1.7)	
Prison and probation service	12 (2.7)	10 (4.7)	2 (0.9)	
Other	16 (3.6)	8 (3.8)	8 (3.4)	
Employment the past 3 months				< 0.001
Full-time or part-time employment	70 (15.8)	26 (12.4)	44 (19)	
Unemployed	180 (40.7)	97 (46.2)	83 (35.8)	
Sick/Activity compensation	133 (30.1)	58 (27.6)	75 (32.3)	
Student	16 (3.6)	2 (1)	14 (6)	
Other	43 (9.7)	27 (12.9)	16 (6.9)	
Primary drug used the past 12 months				1.000
Amphetamine	264 (59.1)	127 (59.1)	137 (59.1)	
Heroin	102 (22.8)	49 (22.8)	53 (22.8)	
Other	81 (18.1)	39 (18.1)	42 (18.1)	
Shared needle, syringe or paraphernalia past month				0.065
Yes	198 (44.9)	103 (49.5)	95 (40.8)	
No	243 (55.1)	105 (50.5)	138 (59.2)	
HBV status at registration (anti-Hbc)^c^				0.375
Positive	5 (1.2)	1 (0.5)	4 (1.8)	
Negative	427 (98.8)	205 (99.5)	222 (98.2)	

^a^Missing data (*n*): Country of birth (98), home region (20), age of debut of drug use (15), age of debut of injection drug use (22), level of education (13), living situation the past 3 months (3), employment the past 3 months (8), primary drug used for the past 12 months (3), shared needle, syringe or paraphernalia past month (9), HBV status at registration (18)

^b^Not applicable

^c^Antibody to HBV core

Underlined numbers representing a *p* value < 0.05

### Drug use

The overall mean age of debut of drug use and debut of injection drug use was 15 years (median 14) and 22 years (median 20), respectively. The most common first drug was tetrahydrocannabinol (THC) representing 282 participants (63.9%), followed by amphetamine representing 77 participants (17.5%). In contrast, the most common first injected drug was amphetamine representing 299 (67.8%), followed by heroin representing 79 (17.9%). Lastly, with regard to primary drug used the past 12 months, 264 participants (59.1%) answered amphetamine, whereas 102 participants (22.8%) reported heroin.

### Mortality

Nineteen participants died during the study period, representing a mortality of 4.2%. Of these, 15 (78.9%) were male and 4 (21.1%) were female. The mean age was 36.8 years, and the cause of death was overdose in 10 cases (52.6%), accident in 1 case (5.3%) and unknown in 8 cases (42.1%). Of the deceased, 8 (42.1%) were HCV positive at NSP registration.

### HCV prevalence

**Fig. 1:**
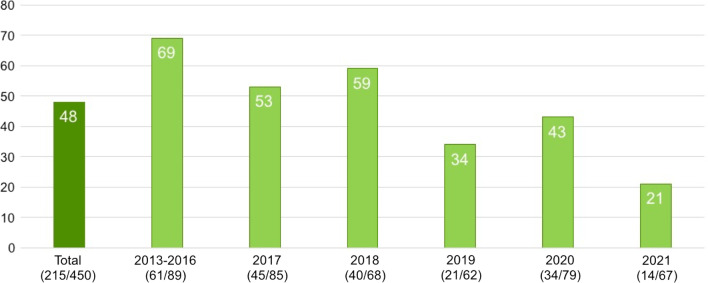
HCV prevalence (%) in total and per registration year. All patients are analyzed based on their registration year, both those positive at registration and those negative at registration and positive at repeat testing

The overall HCV prevalence was 47.8% (215/450) with a declining trend over time, as presented in Fig. 1. Of the 215 HCV-positive patients, 170 (79.1%) had a positive HCV test at registration whereas 45 (20.9%) had a negative registration test and then positive HCV test at repeat testing (5 in 2016, 10 in 2017, 11 in 2018, 4 in 2019, 11 in 2020 and 4 in 2021). 343/450 (76.2%) participants had their NSP registration test taken in Uppsala, whereas 107/450 (23.8%) registered initially at an NSP in another region and had their HCV registration test taken there.

### HCV risk factors

Table 2 displays OR and CI correlated to the outcome positive HCV RNA at registration. The simple univariate regression analysis showed a higher risk of HCV positivity correlated to younger age at debut of drug use (OR 0.927, 95% CI 0.879–0.977), younger age at debut of injection drug use (OR 0.956, 95% CI 0.931–0.982), higher number of visits at NSP (OR 1.004, 95% CI 1.003–1.011) and lower level of education (OR 2.216, 95% CI 1.510–3.251).

**Table 2:** Unadjusted and adjusted odds ratios related to positive HCV status at registration

	Simple univariate regression, *N* = 450^a^		Multiple univariate regression, *N* = 391^b^	
	Odds ratio (95% CI)	*p* value	Odds ratio (95% CI)	*p* value
Gender				
Female	Reference		Reference	
Male	1.045 (0.684–1.598)	0.838	0.774 (0.463–1.293)	0.327
Age at registration	1.012 (0.995–1.028)	0.165	1.025 (1.004–1.046)	0.018
Age of debut of drug use	0.927 (0.879–0.977)	0.005	0.975 (0.916–1.037)	0.423
Age of debut of injection drug use	0.956 (0.931–0.982)	< 0.001	0.963 (0.932–0.996)	0.026
Total number of NSP visits	1.007 (1.003–1.011)	0.001	1.005 (1.001–1.009)	0.027
Level of education				
Upper secondary/Vocational/University	Reference		Reference	
Partial elementary/Elementary	2.216 (1.510–3.251)	< 0.001	1.829 (1.185–2.821)	0.006
Living situation the past 3 months				
All other	Reference		Reference	
Homeless/Shelter	1.377 (0.948–2.000)	0.093	1.348 (0.879–2.067)	0.172
Employment the past 3 months				
All other	Reference		Reference	
Unemployed/Sick/Activity compensation	1.320 (0.873–1.996)	0.188	1.337 (0.832–2.149)	0.230
Primary drug used the past 12 months				
Other	Reference		Reference	
Amphetamine	0.998 (0.607–1.643)	0.995	0.642 (0.870–0.484)	0.642
Heroin	0.996 (0.555–1.785)	0.988	0.973 (0.503–1.882)	0. 935
Shared needle, syringe or paraphernalia past month				
No	Reference		Reference	
Yes	1.425 (0.977–2.078)	0.066	1.233 (0.798–1.904)	0.345
HBV status at registration (anti-Hbc)				
Negative	Reference		Reference	
Positive	0.271 (0.030–2.442)	0.244	0.199 (0.021–1.887)	0.160

^a^Missing data (*n*): Age of debut of drug use (15), age of debut of injection drug use (22), level of education (13), living situation the past 3 months (3), employment the past 3 months (8), primary drug used the past 12 months (3), shared needle, syringe or paraphernalia past month (9), HBV status (18)

^b^Only performed on patients with no missing data in any category

Underlined numbers representing a *p* value < 0.05

When adjusting for possible confounders in the multiple univariate logistic regression, determinants associated with a higher risk of HCV positivity were older age at registration (OR 1.025, 95% CI 1.004–1.046), younger age at debut of injection drug use (OR 0.963, 95% CI 0.932–0.996), lower education level (OR 1.829, 95% CI 1.185–2.821) and higher number of visits at NSP (OR 1.005, 95% CI 1.001–1.009).

### HCV treatment uptake

**Fig. 2:**
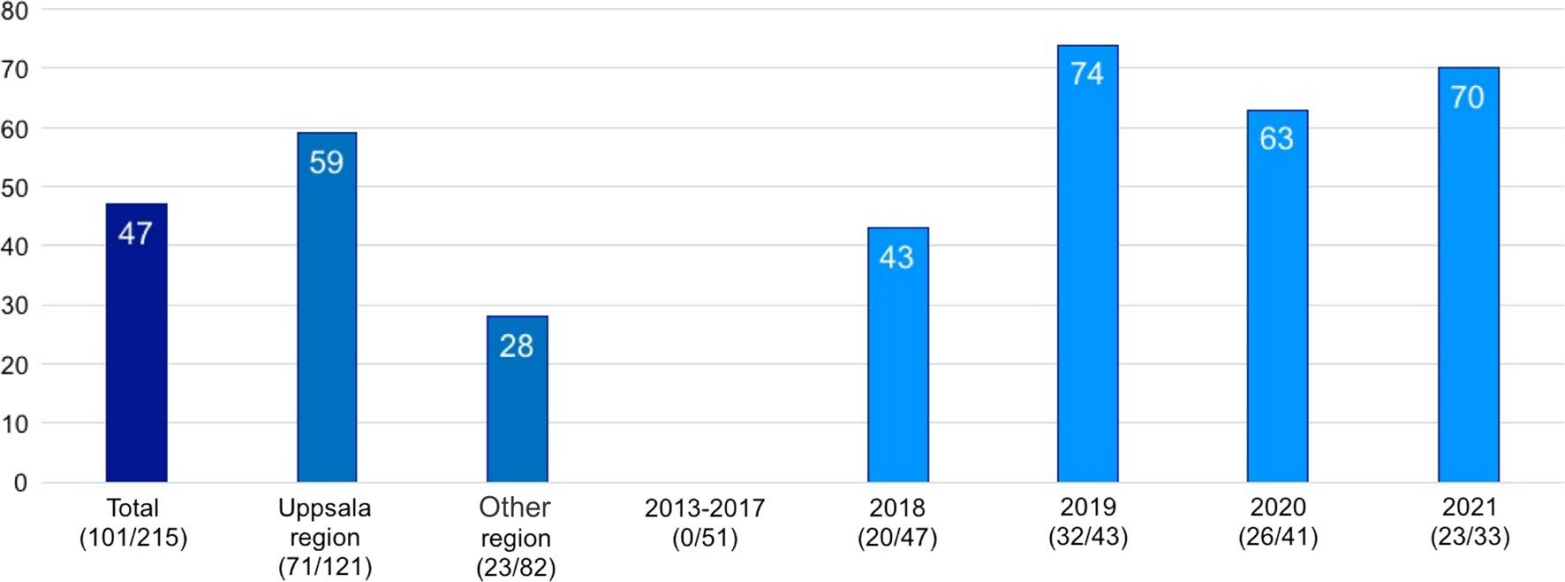
HCV treatment uptake (%) in total, per home region and per year. Treatment year for those treated, year of first positive HCV test for those not treated

HCV treatment uptake is presented in Fig. 2. Out of the 215 HCV positive NSP participants, the proportion who consecutively received HCV treatment were 47% (101/215). After the Uppsala NSP started HCV treatment in 2018, the treatment uptake improved from 0% (0/51) in 2016–2017 to 70% (23/33) in 2021. Those with Uppsala as home region had a higher treatment uptake compared to those with other home region, 58.7% (71/121) versus 28% (23/82).

### HCV treatment outcome

Of those treated, 73 patients (72.3%) were male whereas 28 patients (27.7%) were female. The mean age at treatment was 39. 68 patients (82.9%) were born in Sweden and 14 patients (17.1%) were born abroad. Genotype analysis showed that 51 participants had genotype 1 (50.5%), 44 participants had genotype 3 (43.6%), 4 participants had genotype 2 (4%) and 1 participant had genotype 1 and 3 (1%). In one participant (1%), genotype could not be determined.

Of all 101 patients where treatment was initiated, 78 patients (77.2%) completed the treatment, 11 patients (10.9%) discontinued the therapy and 12 patients (11.9%) were still under treatment at end of study. Excluding those who were still under treatment, this results in a treatment compliance of 87.6% (78/89). Out of the 11 discontinued therapies, 6 were discontinued during treatment, whereas 5 were lost to follow-up (negative HCV RNA at therapy completion, but missing HCV RNA 12 weeks after therapy completion). 77 out of the 78 who completed the therapy had undetectable HCV RNA 12 weeks after therapy completion, indicating a cured infection. One patient had a positive HCV RNA at therapy completion and at follow-up testing 12 weeks after therapy completion. This represents a SVR of 98.7% (77/78).

### Reinfection after HCV treatment

At data extraction, 9/77 patients (11.7%) had gotten a HCV reinfection after therapy completion, of which 6 had started or completed HCV treatment for the reinfection. In the reinfection group, all were male with a mean age of 36.

## Discussion

The HCV prevalence at the first NSP visit among Uppsala NSP participants was 48%, with a declining trend over time. HCV treatment uptake was at 47% and HCV treatment compliance 88%. Of those who completed HCV treatment, the 12-week SVR rate showed an optimal treatment outcome of 99%. The HCV reinfection rate among those treated was 11.7%. The data point out the importance of NSPs providing HCV treatment as well as the need of NSP scale-up and outreach programs to increase access for PWID in order to reach the HCV elimination goal.

### HCV prevalence at the Uppsala NSP compared to NSPs in other settings

The overall HCV prevalence of 48% proved to be lower than both the HCV prevalence projection among PWID in Sweden of 60% and the HCV prevalence shown at the NSP in Stockholm of 62% in 2017 [19, 20]. One possible reason for this could include that NSP participants in Uppsala were younger than in Stockholm, considering that older age could equal an increased number of exposure times for HCV. Conversely, a 2021 Norwegian study showed a very similar HCV prevalence of 42% among people who use drugs [30]. Further data on HCV prevalence among PWID show a range from 16% from NSP data in Australia in 2021, 26% in England in 2021 and 37% in Canada in 2017–2019 [31–33]. The decreasing HCV prevalence over time indicates a reduction of HCV transmission in the PWID community after the NSP opened and HCV treatment started; however, further analysis of the prevalence subgroups is needed to examine this further.

High prevalence of HCV among PWID produces a risk of transmitting HCV through unsafe injection practice. Although the prevalence is lower than earlier Swedish projection data, the studies clearly show that other countries report lower HCV prevalence rates among NSP participants. The HCV prevalence data consolidates that scale-up of low threshold HCV test and treatment opportunities for PWID, such as NSPs, are of importance in Sweden to keep step with the global improvements.

### Improving treatment uptake and compliance

Curing HCV in PWID prevents further HCV transmission and reduces HCV related mortality as well as HCV prevalence. Data from 2019 suggest that 62% of those diagnosed with HCV globally are treated, representing an almost ten-fold increase since 2015 [5]. Compared to the 0.5–2% treatment rate in HCV positive PWID from 2015/2016, our data show a remarkable increase with a total treatment uptake of 47% [19]. By comparison, a NSP report from Australia showed a lifetime treatment uptake among NSP participants of 62% in 2021 [32].

When looking at compliance to HCV treatment, a study among NSP participants in Malmö (Sweden) showed a treatment compliance of 95% in 2018–2019 [34]. A Canadian meta-analysis from 2018 showed that among individuals with recent injecting drug use treatment completion was 96.9%. Both these studies showed somewhat higher rates than our treatment compliance of 88% [35].

To reach the WHO elimination goal, the treatment uptake needs to increase to 90% [25]. Multiple factors can be suggested as underlying causes for absence of treatment or treatment completion in HCV positive NSP participants. For example, participants who only attended the NSP once must return to the NSP on their own to be informed and offered treatment. This is a main obstacle since many NSP participants are lost to follow-up. Similarly, a 2020 Swedish study found a high occurrence of lost to follow-up among patients after HCV diagnosis [36]. Further, patients travelling from neighboring regions may reject treatment due to long distance to the NSP. This hypothesis is strengthened by our data showing a higher treatment uptake for those with Uppsala as home region [37, 38]. Two of the neighboring regions to Uppsala (together comprising half of NSP participants from another region) did not have NSPs in their home region during the study period, highlighting a need of NSPs in these regions. Lastly, the patient must accept and be willing to comply to treatment which may not be a possible choice due to the life situation for some PWID.

To increase treatment uptake and treatment completion PWID access to NSP program, including HCV treatment, is necessary. Additional studies of the HCV positive group not reached with HCV treatment and the PWID group not attending the NSP is needed to find effective ways of approaching this ‘difficult to reach’ part of the PWID population to identify all in need of HCV treatment [39].

### Reinfection rate

A meta-analysis from 2016 showed a 5-year HCV recurrence rate after interferon treatment of 10.67% in the high-risk population, driven mainly by reinfection rather than late relapse [40]. When looking at reinfection among PWID after DAA treatment, data from 2017–2018 show reinfection rates from 3.11 per 100 person years to 8.8 per 100 person years [41, 42]. A 2018 study analyzing HCV treatment of 94 PWID at an NSP in Scotland showed a reinfections rate of 6.5% after 6 months, and 19% after 18 months [43].

Correspondingly, we found a HCV reinfection rate of 11.7% among those treated. This highlights that a high risk of HCV re-exposure prevails among NSP participants in Uppsala. The overall HCV prevalence among PWID must be lowered further through increasing treatment efforts, to decrease this rather high reinfection rate. Further implementation of low-threshold programs (i.e., a further scale-up of NSPs) and outreach programs within current NSPs, such as peer-based ‘secondary needle exchange’ and mobile HCV testing and treatment clinics, are measures that could serve this purpose which have already been implemented successfully in Norway, Denmark and Spain [44–48].

### HCV risk factors

The results show the need for special attention and information on HCV treatment to the subgroups of NSP participants at higher risk for HCV. Older age at registration as a risk factor for HCV most likely illustrates the accumulated need for an NSP over time, as the PWID group have been unaware of their HCV status and therefore also undiagnosed and untreated. Younger age at injection debut as a HCV risk factor is most likely explained by a longer period of injection drug use which equals to an increased number of exposure times, i.e., sharing of unsterile injection equipment. Lower education level is a known factor correlated to higher morbidity and mortality among NSP participants and has been connected to a higher level of injection risk behavior at the Stockholm NSP [12, 28]. In contrast to data reported from the neighboring Stockholm NSP, no association was found between HCV status and female gender, living situation, amphetamine use or younger age in our study.

### Mortality

A Swedish study from 2018, with a mean follow-up time of 13.7 years, among PWID who inject amphetamine showed a mortality of 22%, a mean age of death of 48 years and a majority of fatalities due to external causes (accidents, undetermined intents, suicide and poisoning) [49]. On the other hand, an Australian study from 2018 report a 7% mortality and median age at death of 30.6 years in PWID with high incidence of HCV infection [50]. To further establish mortality data on the PWID group in Uppsala, a longer follow-up period and further examination of the group is needed. This would also be useful for further evaluation of national mortality data among PWID in Sweden.

### Strengths

The standardization of the national quality registry InfCare NSP registration data has enabled the study to include the same information on all participants registered at the NSP since opening, making data comprehensive and thorough. Since the InfCare NSP database is used nationally, registration data from participants first registered at other NSP were available and identical. By following repeat HCV testing results after registration, a longitudinal study design was made possible with information on HCV incidence and HCV reinfection.

### Limitations

Self-reported data include a risk for recall bias as well as social desirability bias as study participants took a face to face survey with NSP staff. When looking at repeat testing all repeat tests were entered, not considering at what NSP the test was taken. Some participants switch NSP intermittently and therefore do repeat testing at different NSP locations. There may be some underreporting on having received HCV treatment in our analysis as some intermittent NSP participants in Uppsala may have received HCV treatment elsewhere. This could possibly cause a risk of underestimation of HCV treatment uptake.

## Conclusions

HCV prevalence, treatment uptake and treatment outcome have improved since the opening of the Uppsala NSP in 2016. However, further measures are still needed to reduce HCV transmission, increase HCV treatment and increase compliance, to reach the global HCV elimination goal. This includes further scale-up of NSPs, efforts to facilitate higher NSP enrolment and exploring and evaluating outreach programs for PWID, such as mobile HCV testing and treatment.

## Data Availability

Data are available from the Needle and Syringe Program quality registry board. Requests can be submitted via email to: Styrgruppen.for.nationellt.sprutbyte.slso@regionstockholm.se.

## Abbreviations

Anti-Hbc: Antibody to HBV core
Anti-HCV: Antibody to HCV
CI: Confidence interval
DAA: Direct acting antiviral
HAV: Hepatitis A
HBV: Hepatitis B
HCV: Hepatitis C
HIV: Human immunodeficiency virus
NSP: Needle and syringe programs
OR: Odds ratio
PWID: People who inject drugs
RNA: Ribonucleic acid
SVR: Sustained virologic response, defined in this report as undetectable levels of HCV RNA in plasma 12 weeks after completed therapy
THC: Tetrahydrocannabinol
WHO: World Health Organization
